# The Tacit ‘Quantum’ of Meeting the Aesthetic Sign; Contextualize, Entangle, Superpose, Collapse or Decohere

**DOI:** 10.1007/s10699-016-9519-2

**Published:** 2017-01-10

**Authors:** Jan Broekaert

**Affiliations:** 1 Center Leo Apostel for Interdisciplinar Studies, Vrije Universiteit Brussel, Pleinlaan 2, 1050 Brussel, Belgium; 2 Department of Psychology, City, University of London, Northampton Square, London, EC1V 0HB UK

**Keywords:** Polysemy, Contextuality, Entanglement, Decoherence, Aesthetic signs

## Abstract

The semantically ambiguous nature of the *sign* and aspects of non-classicality of elementary matter as described by quantum theory show remarkable coherent analogy. We focus on how the ambiguous nature of the image, text and art work bears functional resemblance to the dynamics of *contextuality*, *entanglement*, *superposition*, *collapse* and *decoherence* as these phenomena are known in quantum theory. These quantumlike properties in linguistic signs have previously been identified in formal descritions of e.g. concept combinations and mental lexicon representations and have been reported on in the literature. In this approach the informationalized, communicated, mediatized conceptual configuration—of e.g. the art work—in the personal reflected mind behaves like a quantum state function in a higher dimensional complex space, in which it is time and again contextually collapsed and further cognitively entangled (Aerts et al. in Found Sci 4:115–132, 1999; in Lect Notes Comput Sci 7620:36–47, 2012). The observer–consumer of signs becomes the empowered ‘produmer’ (Floridi in The philosophy of information, Oxford University Press, Oxford, 2011) creating the cognitive outcome of the interaction, while loosing most of any ‘classical givenness’ of the sign (Bal and Bryson in Art Bull 73:174–208, 1991). These quantum-like descriptions are now developed here in four example aesthetic signs; the installation *Mist room* by Ann Veronica Janssens (2010), the installation *Sections of a happy moment* by David Claerbout (2010), the photograph *The Falling Man* by Richard Drew (New York Times, p. 7, September 12, 2001) and the documentary *Huicholes. The Last Peyote Guardians* by Vilchez and Stefani (2014). Our present work develops further the use of a previously developed quantum model for concept representation in natural language. In our present approach of the aesthetic sign, we extend to *individual*—idiosyncratic—observer contexts instead of socially shared *group* contexts, and as such also include multiple idiosyncratic creation of meaning and experience. This irreducible superposition emerges as the core feature of the aesthetic sign and is most critically embedded in the ‘no-interpretation’ interpretation of the documentary signal.

## The Nature of Aesthetic Signs

In this presentation we aim for a model for the dynamics of personal experience related to *aesthetic signs*. We focus in particular on the cognitive representation of contemporary cultural *signs* in the individual’s mind. These descriptions necessarily relate to conceptualizable things—following the perception of which we need here its linguistic and then verbal communication—as inevitable in the presentation of any idea. We realize however with e.g. Schweppenhäuser; “Verbal concepts can never sufficiently describe the complexity of reality: they target the universal and forgo the individual. Aesthetic experience corrects the conceptual description of reality. It refuses to be reduced” (Schweppewnhäuser 2007, p. 253). As a consequence we must do with short descriptions of the aesthetic signs presented here in this paper (specifically Sect. 3) and invite the reader to either look for missing parts of the experience of these signs or tap into personal associations. The ‘quantum’-based concept approach here allows to include contextuality to cognitive dynamics and even has succesfully accomodated measured data from various psychological experimental set-ups (Aerts 2009; Aerts and Gabora 2005; Broekaert 1998). In this paper we will describe the non-classical aspects of aestetics related concept-dynamics and our choice of the specific example signs (Sect. 3) was guided by their proneness to ‘non-classical-logic’ reflection, or the sense of ambiguity after their perception.

We distinguish our use of *context* from Bal and Bryson (1991) where it mainly refers to the ‘framing’ of the art object, with the interpretive status of its historical setting as well as its later circumstantial appearences in the world and its multiplying discourses. Here we understand context centered on the observer, as the variety of mind sets of the observer elicited by the sign. This spectrum of mind sets comes with a spectrum of semiotic codes, personalized and to certain extent idiosyncratic. This focus centered in the observer resonates with quantum theory’s insertion of observer contextuality.

We hold not only this fundamental property of observer contextuality of elementary matter but also entanglement and superposition of states is reflected in the process of thought in general, and commonly in the informediatization and perception of ‘*sign*ificant’ events. The classical experience of art immersion tends to be a long forlorn experience in the current situation, opening-up for ‘creative’–contextual and individualized experience. The canonized experience now makes place for eclectical ambiguity and idiosyncratic re-*creation*.

In a Saussurian perspective context receives a mere linguistic foundation with word differences relating to world differences. The Peircean dynamics of semiosis—in Derrida’s analysis—provides incessantly shifting contexts for the arising of meaning (Bal and Bryson 1991). Therefore, the personal events on life’s trajectory are centered as a stack of codes for present and ongoing understanding. Even more the dualistic separation of sign and context is untenable, not only is the sign polysemous due to multiplicity of contexts, but these contexts themselve need to be submitted to contextualization again. A mental context thus presents a local cognitive environment of more or less coherent meaning, which adheres to the observed sign its significant meaning. The outcome meaning due the personal context in our model is related to ‘connotation’ setting, the semiotic second level meaning as identified by Barthes (1967). Our present approach however distinguishes itself by stating the intrinsic creation aspect of meaning by context, by the irreducible superposition of congruent and incongruent states in the dynamics of the aesthetic sign.

We first render a short presentation of preceding quantum-like modelling of linguistic signs, now well-documented in the literature. For concepts and categories in the expanse of natural language have already been shown to be adequately modeled by quantum-like models. The extended contexts in a personal mind were previously denoted ‘conceptual landscapes’ by Aerts and D’Hooghe (2009), and play a role in the categorization of linguistic concepts. The effect of the context is to collapse the state of the sign into a meaningful concept—an ‘eigen’ state with respect to the given context.^1^ It may seem unnatural to perceive personal cognitive contexts as delineated systems. We do not typically maintain discrete unrelated lines of thinking about a subject, we usually mix aspects and construct meaning in a more fluent organic manner, like subtle variations of ‘conceptual landscapes’. And definitely the intersubjective difference will translate to different meaning patterns when communicated between people. Moreover these conceptual landscapes—the set of relevant and related concepts in a web—change over time within a given person as well. The personal mind changes over time, opinions and insights evolve; transforming the interpretive contexts. The spectrum of contexts is therefor nuanced as notably pointed out very early by Rosh for e.g. colors; naming a color will only approximately convey the impression of the same hue in each person (Rosch 1973).

We hold the perception of the sign entangles with personal contexts, a preparatory state to ensuing reasoning. The collapse to a specific context then engenders its conception within that context. The multiplicity of contexts thus contains the potential of a multiplicity of meaning. The flow of mind concatenates single contexts, and imaginative reflection is made possible by invoking decohering contexts, consecutive collapses bring the sign coherently meaningful into the next context.

Providing a discretized and uniquely identifiable context in this model is thus to certain extent elusive, the availability of contexts is proper to the idiosyncrasy of personal lives. While each context in itself is defined as an identifiable unit, contexts expose the same vague identity as signs themselves. Within the process of the personal mind moreover, the sign may take on the function of the context for further reasoning. This depends on the particular sign; e.g. is it an extended text, or is it itself evolving over a time period.

The unarticulated bodily impact of *aesthesis* in meeting the artistic sign diffuses into the cognitive state of the beholder as they are impregnated by emotions and reflexes besides reflections. The artistic sign pretends to naively invite some oniric relation, while its material presence and spatial or temporal existence naively provoke a realistic immediacy (Hertmans 1999). In the end its core intent is to purposefully engage albeit through an intentionally or unintentionally embedded proneness for multiplicity of codes/contexts. The multiple entanglement to the beholder’s cognitive state creates a unique emotion-dense meaningfull ritchness legitimizing the beholder with the prerogative of creator (hence the ‘produmer’). This variable and idiosyncratic but irreducible superposition is the essential perception of the aesthetic sign.

The entanglement of aesthetic sign and context becomes more complex when the sign pretends to be a documentary or a realistic representation of events. This sign hands a restriction of the interpretive code to the viewer. With narrowed context of observation what interpretive liberty or idiosyncracy remains in viewing the documentary sign? The documentary sign is particular in the sense that the sign also signals a code of ‘no interpretation’. The sign and the reality code are believed to be a joint sign—but the embedded reality code inevitably hits the observer as contextually interpretable.

Our approach to the quantum nature of the sign aligns with Neuman’s view on the semiotic functioning of the polysemous sign (Neuman 2008).

Neuman discusses the polysemy of the sign metaphorically as a superposition of the sign: *the superposition of the sign is defined as the simultaneous existence of mutually exclusive values*. In our approach we formally model the sign by supplying the relevant states as components reflecting different contexts. We do however emphasize the irreducible nature of this composed state of the sign. We hold the state of the superposition reflects the essential nature rather than a mere epistemological decription (of lack of knowledge). The sign, represented by the quantum state takes on pertinent amplitudes each time it hits a conscient context.^2^

The application of quantum-like formalism modeling in psychology, information science, biology, economy and political sciences has now become a tool to handle non-classical behavior and thought of human subjects. This current development in modeling science is now commonly named ‘quantum interaction’ approach—recent applications in social sciences can be found in e.g. Song et al. (2011), Aerts et al. (2000) and Wendt (2015). Following the modeling of concepts we will expose some arguments for quantum-type modeling of signs in the next section, and will put to practice the aesthetic sign afterwards (Sect. 3).

## The Nature of the ‘Quantum-Like’

Before entering our analysis of meeting the aesthetic sign, we must diverge towards the legitimization of the quantum paradigm in our experiential description and, a quantum formalism in our experiental model for this meeting. First of all, the ‘quantum interaction’ approach has not the intention to introduce a pervasive quantum ontology from elementary matter to the cognitive realm (Busemeyer and Bruza 2012; Khrennikov 2010). The natural sciences have developed their optimized methods, theories and ontologies, and continue to update according emerging insights. A more problematic case rests with the interdisciplinary perspective: structures and processes as e.g. auto-poietic systems, patterns, attractors or self-organized systems. These emergent entities have been integrated in our scientific view of reality, their ontological basis or reification is less clear. The extension of the basic physical ontology to an inclusive ontology based on dynamics for life sciences and human sciences have been described by Luhmann (1997) and DeLanda (2006) amongst others. We mentioned in the previous section a typical observation in interdisciplinary research is the finding that many processes are identical in their dynamical form, regardless of variations in the material character of their elements. They are ‘similar’ irrespective of the nature of their substrate. What is the precise value of this observation? The transposition and adaptation of dynamics and phenomena–beyond the metaphorical use as set by Barad (2007)—from one discipline to another, should not reduce the one to the other. Systems of ‘higher’ level—with complex organization—with irreducibility and closure should be acknowledged with a concomitant ontological status of irreducible functionality, nor should it succumb to a reification or a ‘fallacy of misplaced concreteness’. We are then inclined to extend to an inclusive real, informed by the observation process in quantum theory. Something exists if it has ‘a disposition of influencing’. An ‘observer’ should not be considered passive in relation to the sign; the creative aspect of the ‘observation’ puts one more layer to the meaning of the ‘produmer’ (Floridi 2011). Not only classical creation—e.g. information production with respect to lack of knowledge—but ‘quantum’ creation; the emergence of previously non existent features in the meeting of a *sign*.

One possible entrance to the formal understanding of the dynamics of meaning in the human mind is to expose how concepts—expressed by ‘atomic’ terms or words—combine into language. We intend language for interpersonal speech communication and perceiving of signs alike. Previous and ongoing development in the quantum interaction modeling approach exposed non-classical features in the combining of concepts (Aerts 2007, 2009; Aerts et al. 1999; Aerts and Gabora 2005; Aerts and Sozzo 2011; Franco and Zuccon 2010). The core reason for this evolution rests in the more encompassing generality of the non-Kolmogorovian probability structure of quantum theory as opposed to classical probability theory. The experimental revelation of the concept combination problem was demonstrated in experiments by Hampton (1987, 1988). Hampton’s experiments show a deviation from classical set theoretic membership weights. These membership weight were measured on subjects which rated membership of exemplars with respect to concepts, their conjunction or their disjunction. A simple example shows the generality of the problem. At the origin of this research was the observed ‘Guppy effect’ in concept conjunction (Osherson and Smith 1982). Osherson and Smith (1982) proposed the concepts ‘pet’, ‘fish’ and the conjunction ‘pet-fish’ and asked subjects to score the typicality of a Guppy—a small colorful fish, popular in home aquaria—for each of these concepts. It appeared, while the Guppy scored low typicality as a ‘pet’ and low as well for the ‘fish’, it scored a high typicality for ‘pet-fish’. This phenomenon can be considered a flagrant violation of the conjunction of classical logic—the overextension of the conjunction. Allegedly it shows, practical thought ignores the logical fact that the smaller the intersected set, the less probable it is to contain the element. We hold however that this ‘logical fallacy’ rather reveals the combined concepts form a new emergent concept, not logically reducible to its parts. The exemplar sign—Guppy—therefor was partly *created* with respect to the context of combined concepts (Aerts et al. 2012).^3^

Instead of focussing on a categorization problem, Bruza et al. (2009) formalizes a mental lexicon of which the structure is defined by associative links that bind this vocabulary together. While a cultural and social component to these clusters is apparent (Barthes 1967), an extensive idiosyncratic relation network is as pertinent. A fundamental difference between classical and quantum nature comes from their related probability model. Classical probability expresses a ‘lack of knowledge’ of a deterministic underlying reality—the constituent processes that embody the real. On the other hand, non-classical—e.g. quantum—probability expresses an indeterminism in the meeting of an entity and its interacting context. This is a fundamental indeterminism, one that cannot be ‘lifted’ (Aerts 1986). It contrasts the classical ‘discovery’ of the property with the quantum ‘creation’ by observation of the property (Aerts 1998).

This phenomenon of ‘creation by observation’ was identified formally in common situations of reality; e.g. opinion polls. The interviewee’s decision was formally shown to be intrinsically influenced by the context of the inerviewer, thus evidencing a ‘creation’ of the response as was testified by the quantum probability structure (Aerts and Aerts 1995). From the point of view of formal modeling; the spectrum of contexts can be reduced by assigning probability to each of them—giving an impression of ‘averaged’ thinking in a community.^4^ The set of ‘aboutness’-weights obtained from the sign-context modeled thought process thus ‘fingerprints’ a test-group rather than describe the individual original idiosyncratic thought—i.e. personal propensities should not systematically reflect group probabilities. This approach has been previously devised in the quantum concept representation of (Aerts and Gabora 2005), where particular conceptual exemplars are exposed to categorical contexts; e.g. the exemplar *almond* in the contexts of *Fruits*, *Vegetables* and *Fruits or Vegetables*. At present we envisage a semiotic extention of this quantum model for concept representation. We thus envisage the experienced sign as the exemplar ‘input’ and the personal connotations as the distinct contexts. It is clear the associated weigths—of a sign and my spectrum of receiving contexts—are most personal, by time and space enchaining events, coloured by culture and family and the lack of them. These weights are subjective probability amplitudes, they would express a degree of belief or aboutness. Our present approach of representing the sign would thus involve subjective probability— w.r.t. an individual’s state of knowledge—instead of collapse probability derived from group averages. The present approach to the quantum sign thus also targets to use the state representation in Hilbert space using Bayesian probabilities instead of frequentist probabilities. In next section we will develop a qualitative description of some quantum-type contextualizations of aesthetic signs. Since texts, objects and events—signs—can be subject to ‘aboutness’-testing, either related among them as mutual contexts or with respect to codes or interpretational schemes, the processes of the model remain applicable. The concept-based model can thus be extended to aesthetic ‘signs’ in the broadest sense, allowing the essence of the art work to be in the provocation of entangled views and superposed senses. Note that we could devise sign-experiments among a group of viewers that could provide aboutness-measures for an artwork with respect to a number of prescribed mental contexts, which could lead to concensus views but we will adhere to the idiosyncratic pespective here as it validates the aesthetic sign to is most fulfilling personal experience.

Finally we note that it should be taken into account that it may not always be possible to model sign-context representations along the quantum formalism; Hilbert space representation may be too restricted.^5^ The quantum measurement consists in collapsing the state function to an eigenstate of a projection operator of the physical property—an intrinsically probabilistic process. In generalized applications of the quantum theory, these probabilistic weights need to be obtained by statistical analysis of e.g. questionnaires or counts in an internet- or database-search. State functions of a given entity can be decomposed on complete sets of eigenfunctions which can e.g. be associated to ‘exemplars’ in concept-combination testing. In practice this means that a state function of a given entity can be written as a complex sum of eigenstates, each of these terms weighed with a correspondent probability amplitude.^6^

In the next section we discuss the experience of some selected example aesthetic signs using the principles of the quantum contextual model. The processes of meaning entanglement, contextualized meaning collapse, and imaginative decohering, are used for the interpretation of the perception of signs along the quantum model.

## Practicing the Quantum Nature of Aesthetic Signs

Evidently the aesthetic sign has many more dimensions and a larger impact span than the mere linguistic sign as it directly hits the inarticulate experience (Sontag 1961). Our descriptions here cannot stand for the experience itself, ‘by definition’ we are restricted to communicate the sensibly conceptualizable part of experience. However verbally translated perception of the sign is key to our formalised approach here. One should critically admit an overarching discursive context in all the signs described here is their being present in exposition galleries or auditoria—art or academic institutes. This fatally makes the states of the discussed signs prone to collapse in an encompassing context of their being a work of art or a documentary and their related discourses. But even with this constraint the canonic interpretation codes are vague or inexistant, and the idiosyncratic approach is induced.

**‘Mist-room’** by Ann Veronica Janssens is materially an enclosed silent space filled with mist and lit by a few colored ceiling spotlights spaced apart (Janssens 2010). The experience of walking through the silent hued spheres of dry ice mist first silences the mind and raises alertness, the usual mind-spinning and articulate reflection finally stops to let the body breath the slightly moist air, watch the blend of primary colors change en route, roam the silent floor with an occasional human silhouette straying. Then putting all that to an end the mind’s interpreter takes over, the contextual entanglement sets in: ‘why shouldn’t I expect some concreteness somewhere in this room?’, ‘why shouldn’t I question the size of this space and maybe expect labyrintic progression through it instead of my retracing to the entrance’. One questions oneself on being present ‘inside’ the work, literally be part of the installation. Still, while one coincides bodily with the installation, the sphinx-like simplicity of the experience leaves the mind alienated. The reflective exclusion from the art work leaves the produmer ‘in a fog’ (Hertmans 1999). In mist-room the mind quickly decoheres, the mind wanders looking for sensible contexts. Eventually the mind has entangled the sign to idiosyncratic contexts, inter alia: $C_1$–A far echo of the oceanic womb, $C_2$–a mise-en-scene of a Platonic cave, $C_3$–ingredients of an abandoned and strangely muted disco-floor.

The particularity of this sign is—to our opinion—not sensory but reflective deprivation, the mind persists in contextual entanglement and imaginatively decoheres. The overwhelming bodily inclusion however persists the entanglement without resolve or collapse, rejecting the urge to comprehend.

David Claerbout’s projection art work **‘Sections of a happy moment’** roams over perfect stills of a smiling extended family gathered around the youngest one tossing a ball in the courtyard of brand new residential sky-risers (Claerbout 2010). Again this sign slowly entangles the state of mind because to a native-Flemish context—which is proper to the artist—one cannot but read the experience as a paradox. Then surges a context of hidden criticism, of impending disillusionment notwithstanding the elements of a happy atmosphere in utopia. A native-Chinese observer’s context may however read the optimistic version of the work’s title at face value. But the ‘hidden’ context—of which only indicative elements are presented in the work: the spotless modernist building on its tell-tale pilotis. All images are based on photoshopped, rejuvenated photographs of architect Renaat Braem’s ‘Wooneenheid Kiel’—Antwerp, start of building 1951—with Chinese figures pasted in the court yard (Braeken 2010; De Wolf 2010). This manipulation seems to adhere to a critical forewarning about consequences for China’s present-day massive transition to uniform high-rise city living. This ‘echo of the future’ from the present day’s situation of social precarity of ‘het Kiel’ to this modernist architecture is collapsing the mind to the meaning provided by the hidden, but hinted, context. This work did not suffer censorship at the Minsheng art exhibition during the Shanghai World Exposition 2010—the hidden context unperceived or acceptably unknown. The mind is set to contextual meaning, inter alia: $C_1$—the face-value of China’s present-day neoliberal expansion, $C_2$—commercial-type alienation from utopian reality, $C_3$—the present day socially precarious situation of parts of ‘het Kiel’, Antwerp.

The entanglement of the sign to conceptual contexts provides here a potential sting to the art-work. The ‘hidden’ context of political awareness works in both worlds.

The 9/11 terrorist attack, in particular the destruction of the New York WTC and its residents, by-passers and rescue-workers not only reoriented the geo-political agenda, it produced a stream of mediatic visual documents changing global cultural references. One tragically simple documentary photograph by Richard Drew has become known as **‘The Falling Man’** (Drew 2001). The image represents a then unidentified man falling in head down -one leg bent-position, to the abstract background of the vertical lined architecture of the North Twin Tower. A moral resentment refutes an aesthetic reading of the photograph. Between Scylla and Charybdis, the imminent death of this inexorably driven human being is visually fixed in time. Paradoxically, the image is at the border of a culturally acceptable image of ‘dry death’ ( Bataille 1986, p. 56). But shamefully, the image appeals to the aesthetic sense and the picture tends to become an ‘art work’. By these tokens the image is just acceptable to news media, which cannot—by its proper rules—show the true face of war and death. Without showing details, the sign collapses into a meaning context of the horrific. The sign entangles to contexts, inter alia; $C_1$—alienation of time arrested impending death, $C_2$—abstract aesthetic of vertical line pattern with diagonal modulation and accent of aligned figure, $C_3$—visual echo of civilians jumping to their death from “Banzai Cliff” during the final days of the Battle of Saipan, July 1944 (http://www.youtube.com/watch?v=eDUy0uzmaU4.), $C_4$—the loneliness of the drowned ‘refugee girl’ floating in the Mediterranean blue waters in a documentary still of *Drowning for Freedom* (Vice News 2015).

By denying the explicit elements of death, this sign oscillates the mind uncomfortably between mutually excluding aesthetic and moral meaning. This sign tends not to collapse but is repelled to a state of superposition.

The historical colonial expansion followed by recent large scaled (agro)-industrial exploitation have often side stepped and marginalised indigenous cultures into precarious states of subsistence around the globe. Conflicting interests of modernity have arisen in these communities; causing tension—if not amalgamation—between traditional customs and neo-liberal aspirations. The documentary **‘Huicholes. The Last Peyote Guardians’** handles the Huichol community’s struggle to maintain the traditional annual pilgrimage grounds of Wirikuta (Central Mexico)—essential for the survival of their spiritual connection with ‘The Mother’ and their ancestors—in the face of detrimental effects of large scale mining projects and private landownership (Vilchez and Stefani 2014). The documentary by Hernán Vilchez (director) and Paola Stefani (producer) mixes the daily life, the annual pilgrimage, the activism and statements of all stakeholders in a careful balance of interests. This exercise in impartiality seems however to originate from a spiritual quest of the Huichol protagonists and the film director to strive for re-balancing life at Wirikuta—and in extension life on our globe.^7^ Initially the documentary was launched by presenting it during five ‘functions’ (Barnett 2014a, b), reflecting the five cardinal locations of ritual blessing according the Huichol cosmovision. Also during these ‘functions’—the screening of the movie was preceded by Marakame Don José’s invitation towards the audience to join the blessing in the five cardinal directions of the Sun.^8^ In its ensuing showing throughout an impressive number of locations in the Americas and Europe, the screening was always preceded by the Marakame’s blessing ritual, adding to transpose the spirituality of Wirikuta to the viewers in the university auditoria (Perez-Garcia et al. 2016).

The effect of this transposed performance relates to Culler’s description of the semiotic quest for the marked authenticity by ‘the tourist’ (Culler 1990). The viewers veracity quest is fullfilled when the original is recognisably present. The presence and proceedings of Marakame Don José mark the authenticity of the function. The documentary as a visual representation of reality now transforms into fragile reality. The staged material performance enhances the already embedded ‘reality’ code or *no-interpretation* interpretation of the documentary.

The ritual blessing at the screening starts a dynamics in which a coincidence of the signifier and signified is reached: the spiritual ground of Wirikuta has traveled to the screen of the university auditorium—one would reckon. But then again, the documentary learns the true spiritual experience when transported by *hiruki* (peyote) is not disclosed to an ‘external’ viewer and essentially, this is an activist call out to end the inching destruction of their living culture.

We have noted the documentary sign signals a code of ‘no interpretation’, which is challenged by realizing its mere representational relation to ‘the real event’. In the present case the documetary sign is augmented by the staged blessing in the auditorium. The protagonist in the documentary is performing on the stage and empowers the interpretation code of reality. Experiencing the complex documentary sign ‘Huicholes. The Last Peyote Guardians’ preceded by the shamanic blessing, the mind entangles to a succession of shared and less idiosyncratic contexts ; $C_1$—an improptu and unidentified spiritual appeal, alienating an auditorium into a *terra sacra* of sorts, $C_2$—the moral resentment for the pervasive grip of modern life style and its often far reaching and unwanted ecological impact, $C_3$—the disjuncturing dynamics of indigenous outsiderness in a globalized world and their pristine rootedness in their proper world (Appadurai 1990).

Clearly this ‘enhanced’ documentary sign projects its embedded ‘reality’ code with an unexpectedly desambiguating effect. The resulting classical ‘work in context’ configuration keeps the narrative—of positive activism in equilibrium with ‘The Mother’—on track (Bal and Bryson 1991). At first this enhanced sign seems less prone to decoherence and resisting superposition in the oberver’s mindset, but again subject to all personal and idiosyncratic Derridean recontextualizations of (con)text.

## Discussion

We developed the conceptually ambiguous nature of the aesthetic *sign* in terms of a quantum paradigm; its interpretive code elicited by *contextuality*, its resistance to singular reduction by a persisting *superposition* of multiplicity, its dialogueing *entanglement* of partial features to incongruous interpretations, its momentary *collapse* providing well earned insights and its follow-up *decoherence* into enriched ambiguity again.

Our quantum inspired analysis was applied to select aesthetic signs showing these signs are ultimately prone to contextual creation by the produmer—the producing consumer of the sign (Floridi 2011; Toffler 1980). This should be viewed as a true production of an observer outcome, for it is this actualized experience of the sign which is taken onto the subsequent reasoning about and experiencing of the sign. The complex of the artwork, the artistic sign, does enable classical steering of meaning in the beholder, but even then ambiguity is prone to arise idiosyncratically in the mind. Mostly aesthetic signs will diffuse hidden or conflicting contexts.

We also observed that to interact with a sign by entanglement –by means of the personal conceptual landscape—leads to engage in a ‘responsibility’ relation to the real (Barad 2007). To create and change the meaning of a sign will often engage in a moral relation. One could extend this caveat more generally to our engagement with signs in the public sphere: the global expansion of two-way network communication and interaction is ever more altering the potential of individuals, while at the same time the shared *socially* operated signs seem increasingly replaced by idiosyncratic re-created signs (Baudrillard 1970). At the far end of this spectrum, there appears a limitation to idiosyncratic ‘re-creation’ of the sign, e.g. in the false-positive interpretation of pareidolia. It is a collapse to the meaning of an uninvited context idiosyncraticaly provided by a person’s physical embodiment, a personal praxis-driven, non-lingual perception of the sign, but it also signals the solipsistic limitation.

At the short end of the spectrum, the restriction of the interpretive code forwarded by the documentary sign intends to keep this multiplicity of meaning in check, but must ‘fail’ by its proper definition, it remains a representation that cannot be undone by its embedded ‘no interpretation’ interpretive code.

As such, our entire disposition to reflect seems affected by degrees of multiple superposition. In that vein, a quantum approach to psychology has been advocated by e.g. Busemeyer and Bruza (2012) for “[...] fundamental concepts based on quantum formalisms (such as state preparation, measurement, state evolution) and fundamental psychological concepts (such as stimulus, response, information processing)”. The cognitive state on the aesthetic sign can be developed in the same rigorous formal model. Eventually requiring aboutness-data for a configuration of e.g. visual signs and contexts in similar approach as quantum concept modeling (Aerts et al. 2012). This approach also emphasizes the possibility to render personal cognitive dynamics instead of (alleged) group, common or canonic perception dynamics of events. For—we reckon—with respect to meeting the aesthetic sign foremostly the idiosyncratic contexts prevail.

In the semiotics of art, Bal and Bryson (1991) have analysed the multiplicity of the ‘social group’ but also the heterogeneity within them. And commend us that codes are but partially shared and mostly undocumented: “[...]how should we view this immense reserve?” (p. 186). We have advocated that beyond the canon of the codes there is an idiosyncratic reading which lends the sign its strongest personal effect. Finally, the essence of the aesthetic sign is unique in its nature to remain in and sustain the superposition of meaning states and entangles its parts with the ambiguous amalgamation of senses and emotions—akin to some quantum entity.

## Footnotes

1 The simple but non-trivial liar-paradox sentence—‘this sentence is false’—was modeled in the quantum scheme, providing two contexts: $C_1$ ‘the sentence is true’, $C_2$ ‘the sentence is false’. A time-like evolution of the paradoxical true-false succession in the mind of the observer is provided by a Hamiltonian description (Aerts et al. 1999; Broekaert et al. 2006).

2 There is no question of soliciting Everett’s parallel universa interpretation to cope with the measurement problem of collapsing to a single eigenstate.

3 The definition of ‘classical’ in our model relates to the behavior in measurement or observation. An entity, is a ‘classical’ entity if for any of its states and for an arbitrary measurement one can predict with certainty which will be the outcome of the measurement. For quantum entities this will obviously not be satisfied. In these quantum based models we use a ‘probabilistic’ reasoning, and refer to ‘classical’ entities if the probability structure of the statistics is Bayesian and refer to ‘quantum’ entities otherwise.

4 In principle the quantum mathematical formalism in Hilbert space is summarized to the states, the context projectors and associative weights. Let $\{C_i\}$ be the set of contexts, the instant master context for a given group is $\sum _i p_i C_i$ with $\sum _i p_i =1$. Where probabilities $p_i$ are obtained in ‘aboutness’-score testing in a community.

5 It may not always be possible to consistently fit the experimentally obtained weights or aboutnes-scores. In particular cases an extension to quantum *field* theory, using Fock spaces, can provide state functions which fit these weights (Aerts 2007). Other non-quantum generalizations like SCOP—state-concept-property—descriptions extend data fitting applicability (Gabora and Aerts 2002).

6 Let the state of the sign be described by $\varPsi = \sum _i w_i \phi _i$ with $\sum _i \vert w_i\vert ^2 =1$. The application of the projector $P_i$ proper to the context $C_i$ will collapse the wave-function $P_i \varPsi = w_i \phi _i \ \ \rightarrow \ \ \varPsi |_\mathrm{after \ measurement} = \phi _i \nonumber \ \mathrm{with\ probability} = | w_i |^2$.

7 An interview on the making and context of the documentary was taken from Hernán Vilchez and Don Jose Ramírez by drs. Lorena Perez-Garcia and the author at Leiden University on 18/05/2015.

8 A Huichol elder, traditional healer and wise man is called ‘Marakame’ in Huichol language.
